# Nineteen Cases of Sudden Death

**Published:** 1908-09-12

**Authors:** 


### SPECIAL ARTICLES.

### NINETEEN CASES OF SUDDEN DEATH.

The question of sudden death is of such importance to every medical man that the following illustrative examples may be of service. They are consecutive cases from a single poor-law district, and so afford a rough idea of the relative frequency of some of the causes of sudden death. There were in the same period, and in the same district, altogether 1,000 deaths, mostly due to diseases that had been diagnosed before death; out of these 1,000 deaths from all causes the following 19 were cases of sudden death, by which we mean death supervening within fifteen minutes of the beginning of the fatal seizure in persons who have been at their usual avocation up to the last moment, who are then suddenly taken ill, and are dead by the time the doctor arrives. They are the kind of cases which the general practitioner is called upon to examine post-mortem. It is obvious that, amongst only nineteen cases, it is not possible for every cause of sudden death to be represented, but since they are consecutive and unselected it is probable that the commoner varieties will occur in the series. The cases are not set down in the order in which they occurred, but rather in groups so that similar cases come together.

### A.—Cases of Sudden Death due to Cerebral Lesions.

Case 1. *Ordinary Apoplexy.*—A male, aged 58, had a stroke six years before his death; at the time of that stroke he had partial hemiplegia, but from this he gradually recovered. He had done no active work since, but was up and about, and in good general health until the day of his death. On the forenoon of that day he went to the water-closet, and was heard to fall on to the floor. Assistance reached him at once; he was speechless, his countenance assumed a leaden, sub-livid hue, and coma rapidly advanced. The right arm was, with a faltering, ill-directed action, carried to the head. Respiration was slow and heavy without much stertor, the intervals between the efforts gradually increasing. The pulsations at the heart and wrist were full and powerful, and continued most distinctly for some time after respiration ceased. The pupils were at first contracted, and afterwards somewhat, but not greatly, dilated. Death took place in about eight minutes.

At the autopsy a very extensive cerebral hæmorrhage was found, which filled the ventricles, disorganised a large portion of the cerebrum, and extended under the pia mater all over the base of the brain. There was advanced chronic Bright's disease in the kidneys.

Case 2. *Superficial Cerebral Hæmorrhage.*—A male, aged 56, formerly a smith, had not followed his avocation for four years owing to paresis of his left arm and leg; he had enjoyed good health, however. He had had to make prolonged efforts during micturition for some while past. On the day of his death he went to a urinal, and whilst micturating was observed to glide easily to the ground; he gave two or three short cries, accompanied by long-drawn inspirations, but there was no struggling. Within two minutes after he fell he ceased to breathe, but the heart continued to beat, forcibly at first, and afterwards in a series of three or four faint pulsations at gradually lengthening intervals, for nearly three minutes. The face was not remarkably livid; the pupils were moderately dilated. Death took place in between four or five minutes.

In this case an extensive superficial hæmorrhage was found covering a large portion of the brain. The fourth ventricle was the only part of the interior in which effused blood was present. There was no organic disease other than atheroma of vessels.

Case 3. *Multiple foci of Cerebral Softening.*—A male, aged 71, was found dead in bed at 4 a.m., having retired to rest at 8 the preceding evening, to all appearances in his usual health. At autopsy the brain showed no effusion of blood anywhere, but in several parts of its interior, particularly in the white matter of the cerebrum, in the corpora striata, optic thalami, and crura cerebelli, there were many little irregular pits of ochrey colour, closely resembling in form and size those seen on cutting open a Stilton cheese. All the cerebral arteries were very atheromatous, with calcareous patches; they were quite full of clotted blood firmly moulded within them.

The whole *aorta* was rigid and inelastic from atheromatous and calcareous changes; the *heart* seemed healthy, except for atheroma of the aortic valves; the *kidneys* were smaller than usual, and granular upon the surface, but not more so than is almost the rule in men of this age.

Case 4. *Cerebral Syphilis.*—A female, aged 28, had suffered for many years from the effects of syphilis. There had been caries of the frontal and right parietal bones, and on several occasions she had had general convulsions, sometimes with, sometimes without, loss of consciousness. On the evening of her death she seems to have had a very severe fit when in bed at 9 p.m.; deep coma supervened, and she died in a few minutes.

The brain was small and atrophied; it was the seat also of gummatous infiltration. No other organ was affected.

### B.—Cases of Rupture of an Aneurysm.

Case 5.—A female, aged 74, was in bed at 10 p.m., whither she had retired two hours before, apparently in her usual health. Her bed-fellow found her make a sudden attempt to get out of bed, and fall back during the effort. She was dead almost instantaneously.

A small saccular aneurysm just above the aortic valves had ruptured into the pericardium, which contained about twenty ounces of blood.

Case 6.—A male, aged 59, a weaver, of spare frame, medium height, and temperate habits, took a short walk, and on his return was practising upon the violoncello, previous to playing it at church, when he was seized with intense pain across the forehead, vomiting, and faintness. He sat with his body bent forward, the face pale, shrunk, and expressive of agony, the pupils slightly dilated. The intellect was at first unimpaired, though tne pulse was scarcely perceptible; he soon experienced intense pain in the cardiac region, became unconscious, and died.

A recent dissecting aneurysm of the ascending aorta was found, which had ruptured into the pericardium.

Case 7.—A male, aged 35, a tall, well-developed, stout young man, had been very intemperate in his habits, but had also worked hard in a factory. He had recently complained of obscure pains in the back, but had not seemed extremely ill. When in bed one evening he was heard to groan, and was immediately after seen to turn on to his left side. He died within a few minutes.

It was found that an aortic aneurysm had partially eroded the third dorsal vertebra, and then burst into the left pleural cavity.

C.—Cases of Sudden Death Due to Degeneration of the Myocardium.

Case 8.—A male, aged 54, a tailor, ate a hearty breakfast at 8 a.m., having previously worked upon his shop-board for two hours with his accustomed energy. After the meal he returned to his work in the usual cross-legged posture, then went to the water-closet, returned to his work again, and a few minutes afterwards spoke of pain in the head; he immediately fell from his seat dead.

The brain was examined with minute care, but no lesion could be found. The veins and sinuses were enormously distended with blood, as were also the arteries. It is just possible that there may have been an embolism, but no embolus was found, nor was there any source for one discovered; moreover, the cerebral arteries as well as the veins were full of blood, so that, notwithstanding the pain in the head he had complained of, it seemed more likely that the congestion was due to failure of the heart. The latter was thin walled, and the muscle was friable; there was extensive atheroma of the aortic valves, but no incompetence or stenosis; the coronary arteries were also atheromatous, and this seemed to be the likeliest cause of the poorness of the heart muscle. Neither the kidneys nor any of the other viscera were diseased. The stomach contained a large undigested meal, consisting chiefly of porridge.

Case 9.—A female, aged 73, a tall, hearty, old woman, was sitting upon the water-closet, to which she had walked unassisted, when she suddenly called out that she was blind. She was helped into a chair; there was no struggling; in less than ten minutes she was dead, the pulse ceasing rather before the last inspiration was heard. Upon inquiry it was found that she had complained of palpitation for two or three days before death.

No cause for death existed, except the condition of the heart, which was the seat of extensive fatty infiltration. Around the upper attachment of the peritoneum was a complete collar of fat, one to three inches deep.

Case 10.—A male, aged 71, a robust-looking, stout man, had been subject to winter cough for years, but was otherwise in pretty good health. On a winter's day he ate heartily of porridge and milk in a warm room, and immediately afterwards went out into the cold air, walking with alacrity across an open yard; suddenly he "lost his breath," and, seizing some railings, glided to the ground. He was carried indoors. The doctor arrived two minutes later, but found life extinct. The face was livid, the pupils a little dilated. The cause of death was acute dilatation of a fatty right ventricle, following the exposure to cold and exercise after a full meal.

Case 11.—A male, aged 56, knew that he had a thoracic aneurysm; being very depressed at this, he attempted to commit suicide. He used an old rusty shoemaker's knife, and tried to cut his throat with it. The knife was so blunt that the skin was only excoriated, and no blood had flowed; he was heard to fall, and his friends rushed in and found him still breathing, but he was dead a moment or two afterwards.

A large aneurysm of the ascending aorta filled nearly the whole of the central part of the chest. Portions of the sternum and of three of the vertebræ were absorbed by the tumour, which was making its way behind the right clavicle. No part of the sac had given way.

The cause of death was undoubtedly syncope, caused by the excitement under which the man was labouring.

D.—Other Cases.

Case 12.—*Sudden Death Due to Valvular Heart Disease.*—A female, aged 45, died unobserved during the night. She had not been under medical treatment, and had made no complaint. She ate her supper as usual, and went to bed apparently in good health. She was found dead next morning; a little blood was oozing from her mouth on to the bed clothes.

The cause of death was an unsuspected mitral stenosis of old standing. Pulmonary apoplexies accounted for the blood which escaped from the mouth.

Case 13.—*Mediastinal New Growth Obstructing the Air Passages.*—A female, aged 67, was in the act of dressing herself one morning when, suddenly becoming faint, she sank into a chair and expired in a few minutes. It was in the winter, the morning was cold, and there was no fire in the room. She had apparently been in quite good health hitherto, though on inquiry it was found that she had complained of some dyspnœa the day before her death.

The cause of death was asphyxia from obstruction to the air-passages, due to a large mass of mediastinal growth, together with dilatation of the right ventricle. The lower part of the trachea and both the bronchi were much compressed and flattened.

Case 14. *Persistent and Very Large Thymus Gland.*—A male, aged 10, was found dead in bed one morning. He had been feeble-minded; he was put to bed in his usual health the night before his death; he had never been the subject of epilepsy, nor had he been suffering from any disease requiring medical treatment.

Post-mortem, the brain was very small, which was to be expected seeing that he was an idiot; there was no other morbid lesion within the cranium. The thymus gland was of great size, mottled, with intense congestion and minute spots of extravasated blood. The thyroid gland was also larger than normal. The suprarenal capsules were small, each weighing less than half a drachm. The remaining viscera were all healthy to the naked eye. It was clear that the thymus gland, particularly if it should from any cause become more turgid than usual, must from its great size have materially compressed the great blood-vessels and breathing tubes in the upper part of the chest. Death was in all probability due to asphyxia. The condition is what the Germans style "Thymus-Tod," and is closely related to the "status lymphaticus," which has been much talked about of late.

Case 15. *Acute Phlegmonous Œdema of the Glottis.*—A male, aged 69, a hale, powerful old man of large frame, and generally good health, was suddenly seized with acute suffocative dyspnœa early one afternoon. A doctor was called in at once, but from the moment of his seeing the patient until his last inspiration scarcely a minute elapsed. A feeble pulse was twice perceived, but the inspiratory movements had ceased. The countenance was pale and cyanosed; the pupils were of natural size; the jugular veins were enormously swollen, like great cords passing down the neck. It appeared that this old man had been out for a holiday the day but one before his death, and came home in his usual health. He had first complained of slight indisposition on the morning of his death, and had consequently remained in bed. The moment of seizure was carefully observed; and from the concurrent testimony of many it is clear that not more than two and a-half minutes intervened between that time and death.

The immediate cause of death was in the larynx: the epiglottis was erect, its apex and sides greatly swollen and œdematous; at its base on either side were many pale yellow purulent foci in the cellular tissue, and there were similar foci over the inner surfaces of the arytenoid and thyroid cartilages; in the glottis itself there was so much œdema that the passage was completely occluded, serous fluid flowing out on puncturing the mucosa. The kidneys and other organs showed no changes of importance. The condition was one in which the only possibility of saving life was instantaneous tracheotomy, as in the similar cases described by Sir Felix Semon.

Case 16. *Sudden Hæmatemesis.*—A male, aged 24, had led a very dissolute life; but although pale and sickly in appearance his general health was good, and he was well nourished. He had been doing his ordinary work one morning; and then, after mid-day dinner, he was carrying a heavy box up a flight of steps. He suddenly noticed blood in his mouth, and immediately there was profuse hæmatemesis. He was dead in two minutes, the last efforts at inspiration being accompanied by a deep purple congestion of the face. Almost the last indication of life was a general epileptiform convulsion.

The liver was extremely large and firm, in a condition of hypertrophic cirrhosis. The kidneys and spleen were also hypertrophied. There was no lesion in the stomach, lungs, heart or brain; the blood seemed to have come from ruptured varicose veins at the lower end of the œsophagus.

Case 17. *Hæmoptysis.*—A male, aged 33, tall and well made, was known to have phthisis, but was not laid up with it. He was straining at stool and coughing, when he began to spit blood rapidly; the hæmoptysis continued, and in about twelve minutes he was dead, dying of suffocation rather than from loss of blood.

The only diseased organs were the lungs; these were the seat of extensive phthisis; in the wall of a large irregular vomica in the left upper lobe there was an exposed pulmonary arteriole, not occluded by clot; and from an opening in this the fatal hæmoptysis had occurred. The bronchi and trachea were almost completely full of blood.

E.—Cases in which No Adequate Cause for Sudden Death was Discovered at the Autopsy.

Case 18. *History of Convulsions. No Lesion Found.*—A male, aged 44, was found dead in bed. He was a short fat man, who for two years previously had been the subject of severe epileptiform fits, which were often succeeded either by maniacal excitement or by quiet incoherence, which passed off in a day or two. He went to bed after his evening meal, apparently in his ordinary state of health. In the morning he was found dead, the face very much congested.

All the organs were carefully examined, but no obvious cause of death could be found. The brain itself was healthy, but the pia-arachnoid membranes covering the cerebrum were opaque, and there was excess of subarachnoid fluid. There was a small concretion in the vermiform appendix, but no evidence of recent appendicitis. The heart, lungs, air-passages, kidneys, and other organs were all natural.

Case 19. *Sudden Death in the Night. No Adequate Cause Found.*—A male, aged 12, during the night before death, having previously been in good health, rose from bed two or three times and vomited; sleeping, however, during the intervals. Next day he seemed to be in fairly good health, though he was listless. Next night those sleeping in the next room heard him breathing hurriedly, but they did not go to him. Two hours later he was found dead in bed. The limbs were rigid, frothy saliva had flowed down the chin, and the fingers were firmly flexed with the thumb opposed, not to the palm, but to the forefinger.

The brain showed no abnormality, notwithstanding a very careful examination of every part of it. None of the organs showed any pathological changes; the only point of note, though probably not connected with the cause of death, was that eight specimens of ascaris lumbricoides were found in the small intestine.

In conclusion, we would point out once more that cases of sudden death as a termination of diseases which are under treatment are excluded from the above; so that sudden deaths from such causes as embolism after operations, and so forth, do not come in. Sudden deaths from external causes, such as accidents, cut throat, murder, bullet wounds, and poisons, are also excluded. The nineteen cases are consecutive, but we would call attention to the absence of representative cases of some other not uncommon causes of sudden death, particularly (1) pontine hæmorrhage, (2) rupture of a thoracic aneurysm into a bronchus, (3) aortic incompetence and (4) Addison's disease.

